# Mechanisms and functional implications of the degradation of host RNA polymerase II in influenza virus infected cells

**DOI:** 10.1016/j.virol.2009.10.003

**Published:** 2010-01-05

**Authors:** Frank T. Vreede, Annie Y. Chan, Jane Sharps, Ervin Fodor

**Affiliations:** Sir William Dunn School of Pathology, University of Oxford, South Parks Road, Oxford OX1 3RE, UK

**Keywords:** Influenza virus, RNA-dependent RNA polymerase, RNA polymerase II, Transcription, Gene expression, Ubiquitylation

## Abstract

Influenza viruses induce a host shut off mechanism leading to the general inhibition of host gene expression in infected cells. Here, we report that the large subunit of host RNA polymerase II (Pol II) is degraded in infected cells and propose that this degradation is mediated by the viral RNA polymerase that associates with Pol II. We detect increased ubiquitylation of Pol II in infected cells and upon the expression of the viral RNA polymerase suggesting that the proteasome pathway plays a role in Pol II degradation. Furthermore, we find that expression of the viral RNA polymerase results in the inhibition of Pol II transcription. We propose that Pol II inhibition and degradation in influenza virus infected cells could represent a viral strategy to evade host antiviral defense mechanisms. Our results also suggest a mechanism for the temporal regulation of viral mRNA synthesis.

## Introduction

Viruses have developed a wide range of mechanisms to inhibit the expression of host genes during the process of virus replication (Lyles, 2000). Although this might provide the virus with higher levels of cellular resources such as nucleoside triphosphates to be used for the biosynthesis of viral gene products, it is now believed that the major role of virus-induced inhibition of host gene expression is to inhibit antiviral host response. Viruses can interfere with various steps in host gene expression—transcription, RNA processing and transport, and translation. For many RNA viruses, the host transcriptional apparatus represents a logical target for inhibition of host gene expression as they replicate in the cytoplasm of the host cell without any obvious requirement for host transcriptional activity. Influenza viruses are an exception, since they replicate in the nucleus of the host cell and require an active host RNA polymerase II (Pol II) (Amorim and Digard, 2006; Engelhardt and Fodor, 2006). In particular, influenza virus cannibalizes host Pol II transcripts to produce RNA fragments that are needed to initiate viral mRNA synthesis (Bouloy et al., 1978; Krug et al., 1979). Moreover, ongoing Pol II transcription is required for the nuclear export of viral mRNAs (Amorim et al., 2007). Therefore influenza viruses are absolutely dependent on active transcription by host Pol II for their replication and indeed, inhibitors of Pol II, i.e. α-amanitin or actinomycin D, block influenza virus replication (Lamb and Choppin, 1977; Mark et al., 1979).

However, recently our group showed that the influenza virus RNA polymerase complex, a heterotrimer of three subunits, polymerase basic protein 1 (PB1), polymerase basic protein 2 (PB2), and polymerase acidic protein (PA), binds to the C-terminal domain (CTD) of the large subunit of initiating Pol II and proposed that this interaction leads to the inhibition of Pol II activity in influenza virus infected cells (Chan et al., 2006; Engelhardt et al., 2005). We hypothesized that on one hand, hijacking the host transcriptional machinery might allow the virus to gain access to factors, e.g. capped RNA fragments, splicing factors, and factors for the assembly of viral mRNPs, on the other, it could contribute to the inhibition of host gene expression which inevitably would affect genes involved in antiviral host responses. Therefore, the ability of the viral RNA polymerase to inhibit Pol II might be a significant factor in viral pathogenesis.

Indeed, the RNA polymerase has been shown to be an important determinant of influenza virus pathogenicity (Naffakh et al., 2008). Several mutations in the RNA polymerase genes have been described that contribute to the increased pathogenicity of influenza viruses in various in vivo model systems; for example, it is now believed that the trimeric RNA polymerase complex of the 1918 pandemic strain was a significant contributor to the unusually high pathogenicity associated with this viral strain (Tumpey et al., 2005; Watanabe et al., 2009). The RNA polymerase genes were also found to contribute to the high virulence of the human H5N1 influenza virus isolates (Salomon et al., 2006). A more efficient RNA polymerase could lead to viruses with increased replication potential which could efficiently outcompete and escape the host innate immune responses (Grimm et al., 2007). However, a more direct role of the viral RNA polymerase in determining pathogenicity has not been excluded. For example, the RNA polymerase might be involved in virus–host interactions directly leading to the inhibition of the expression of antiviral host genes or in a general inhibition of host gene expression.

The association of the viral RNA polymerase with the host Pol II transcriptional machine is well documented (Engelhardt et al., 2005; Mayer et al., 2007; Rameix-Welti et al., 2009), but the implications of this interaction for the functionality of Pol II remain to be understood. Recently, Rodriguez and colleagues reported that influenza virus infection causes a specific degradation of the large subunit of Pol II and suggested that the proteolytic activity of the PA subunit of the viral RNA polymerase was involved (Rodriguez et al., 2007). Here we investigated the effects of the association between the influenza virus RNA polymerase and Pol II on host transcription further. We found that the binding of the viral RNA polymerase to the initiating form of Pol II induces its inhibition possibly via triggering ubiquitylation and proteasome-mediated degradation. We propose that the viral RNA polymerase-mediated inhibition of Pol II plays an important role in inhibiting host gene expression and consequently, in inhibiting antiviral host response. This might have important implications for viral pathogenesis.

## Results

### Influenza virus infection induces degradation of Pol II

**Fig. 1:**
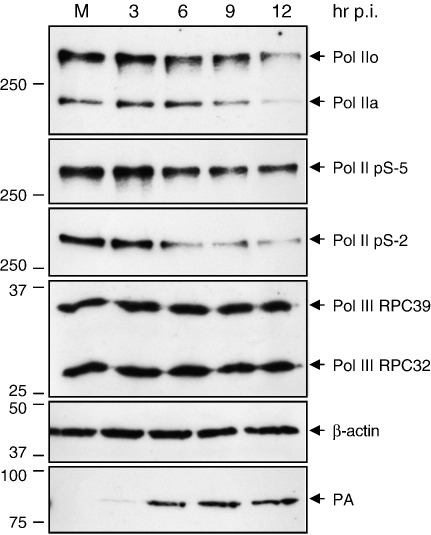
The large subunit of Pol II is specifically degraded in cells infected with influenza virus. 293T cells were infected with influenza A/WSN/33 virus or were mock-infected (M). Cell lysates were prepared at the indicated time points post infection and analyzed by Western blotting. The antibodies used to detect the non-phosphorylated (Pol IIa) and phosphorylated (Pol IIo) forms of Pol II, serine-5 phosphorylated Pol II (Pol II pS-5), serine-2 phosphorylated Pol II (Pol II pS-2), the RPC32 and RPC39 subunits of Pol III, β-actin, and the viral RNA polymerase subunit PA are specified in the Materials and methods section. The identity of bands is indicated on the right. Size markers in kDa are indicated on the left.

Our group reported previously that the influenza virus RNA polymerase complex binds to the CTD of the serine-5 phosphorylated form of Pol II at 3 h post infection (Engelhardt et al., 2005). The CTD of Pol II represents a landing pad for numerous host factors involved in host mRNA processing (Hirose and Manley, 2000; Howe, 2002; Proudfoot et al., 2002) and we hypothesized that its association with the viral RNA polymerase could affect Pol II function. To address this, we infected human 293T cells with influenza A/WSN/33 virus, harvested cells at 3, 6, 9, and 12 h post infection, and analyzed Pol II by Western blot using an antibody recognizing both the non-phosphorylated transcriptionally unengaged (Pol IIa) and the phosphorylated transcriptionally engaged (Pol IIo) forms of Pol II (Fig. 1). We found a significant reduction in both forms of Pol II at late points post infection. In particular, we observed a reduction in the Pol IIo form from 6 h post infection. In comparison, there was a slight delay in the reduction of the Pol IIa form with a clear reduction from 9 h post infection.

Next we analyzed the same cell lysates with antibodies that specifically recognize the serine-2 and serine-5 phosphorylated forms of Pol II. Serine-5 phosphorylation of the CTD is characteristic of the initiating form of Pol II, engaged in capping, while serine-2 phosphorylation is prevalent in the CTD of the elongating form of Pol II (Palancade and Bensaude, 2003). We found a clear reduction in both the initiating and elongating forms from 6 h post infection with a more pronounced effect on the elongating form (Fig. 1). We have also analyzed β-actin and two different subunits of host RNA polymerase III, RPC32 and RPC39, none of which showed any detectable reduction (Fig. 1), suggesting that the effect on Pol II was not due to a general proteolytic degradation of host proteins in influenza virus infected cells. A Western blot analysis of the PA subunit of the influenza virus RNA polymerase complex confirmed that the cells were infected (Fig. 1). Taking into account that the half-life of the large subunit of Pol II is about 12–16 h (Goldberg and St John, 1976; Rodriguez et al., 2007) (Chan and Fodor, unpublished), these results show that there is a specific degradation of the large subunit of Pol II at late points post infection in cells infected with influenza virus.

### Viral infection results in the reduction of Pol II engaged at the promoter region of Pol II genes

**Fig. 2:**
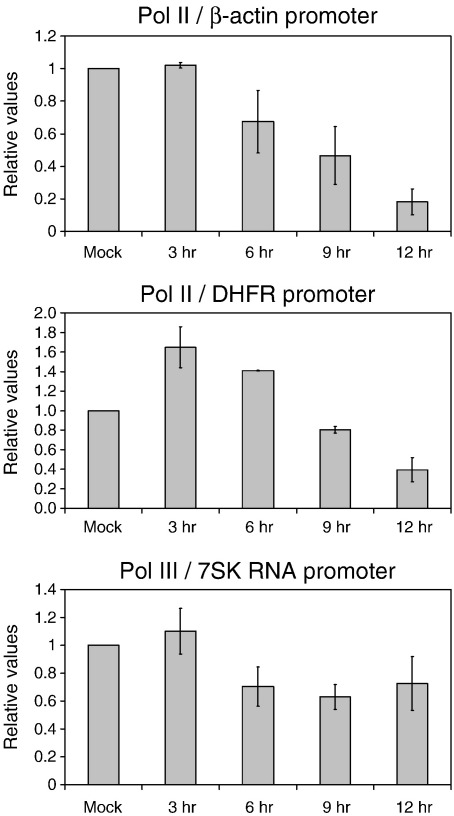
Analysis of the association of Pol II and Pol III with promoter DNA during the viral life cycle by using ChIP. ChIP was performed using lysates from 293T cells either mock-infected (M) or infected with influenza A/WSN/33 virus for the indicated periods of time and antibodies against Pol II (N-20) or Pol III (C32-3). Quantitation was performed by real-time PCR using primers specific for the promoter region of the β-actin, DHFR, or 7SK RNA genes as described (Chan et al., 2006). Polymerase densities were expressed relative to the mock sample which was set to 1. An average of data from two independent experiments is shown with range.

Previously, our group reported that influenza virus infection inhibits Pol II elongation (Chan et al., 2006). In particular, it was found that there was a significant reduction in Pol II densities in the coding, but not the promoter region, of the DHFR and β-actin genes in influenza virus infected cells compared to mock-infected cells at 3 h post infection. Having observed that the large subunit of Pol II is degraded in infected cells, we performed a chromatin immunoprecipitation (ChIP) assay to examine the densities of Pol II at the promoter region of the DHFR and β-actin genes during the viral life cycle (Fig. 2). We found no change in Pol II associated with the β-actin promoter at 3 h post infection. In contrast, at 6, 9, and 12 h post infection, there was a reduction. Interestingly, for the DHFR promoter we observed a transient increase in Pol II at 3 h post infection, followed by its gradual reduction at later time points. These results are consistent with the observations above that Pol II is degraded in influenza virus infected cells at late points post infection. As a control, we have also analyzed the association of Pol III with the 7SK RNA promoter (Fig. 2). We found no effect at 3 h post infection although at the 6 h time point there was an apparent reduction. However, in contrast to Pol II, no further reduction was observed at 9 and 12 h suggesting that influenza virus might affect Pol II and Pol III function differentially.

### Mechanism of Pol II degradation in influenza virus infected cells

**Fig. 3:**
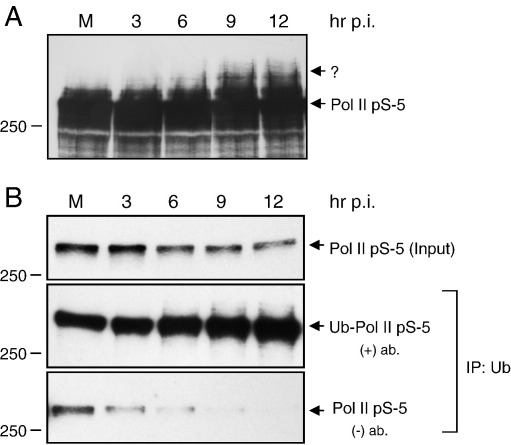
Ubiquitylation of the large subunit of Pol II increases during the viral life cycle. (A) Overexposure of the serine-5 phosphorylated form of Pol II (detected with the H14 antibody) from mock (M) or influenza A/WSN/33 virus infected cells (harvested at the indicated time points post infection). High molecular weight bands recognized by the Pol II-specific antibody, apparent at the 6-12 h time points, are indicated by “?”. (B) Immunoprecipitation of ubiquitin from lysates of mock or virus infected cells. Input samples (upper panel) and immunoprecipitates with (middle panel) or without (lower panel) ubiquitin-specific antibody were analyzed by Western blot using the H14 serine-5-specific Pol II antibody.

Having determined that the large subunit of Pol II is specifically degraded in influenza virus infected cells, resulting in the decrease of transcriptionally engaged initiating Pol II at the promoter region of Pol II genes, we were interested in establishing the molecular mechanisms of Pol II degradation. Interestingly, overexposure of Western blot analyses of the initiating form of Pol II from influenza virus infected cells revealed high molecular weight Pol II-specific signals at late points post infection (Fig. 3A). These results suggested that Pol II could be a substrate for secondary modifications, i.e. ubiquitylation, leading to an increase in its molecular weight. To test this hypothesis, we performed immunoprecipitation of influenza virus infected cell lysates with a ubiquitin-specific antibody (Fig. 3B). Western blot analysis of the immunoprecipitates with a Pol II-specific antibody showed that increasing amounts of the ubiquitylated initiating form of the large subunit of Pol II were present late in infection. Taken together, these results suggest that influenza virus infection results in an increase in the ubiquitylation of Pol II or, alternatively, Pol II is present in complexes containing ubiquitylated proteins. However, the observed increase in the mobility of the large subunit of Pol II (Fig. 3A) suggests that the large subunit itself is a substrate for ubiquitylation. Thus, influenza virus infection results in Pol II ubiquitylation possibly leading to its degradation by the proteasome pathway.

### The role of the viral RNA polymerase in the degradation of Pol II

**Fig. 4:**
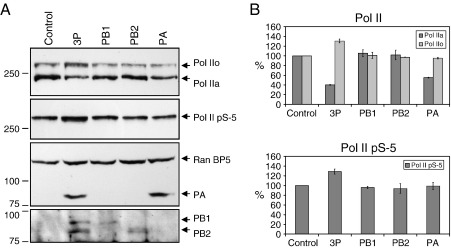
Expression of the trimeric viral RNA polymerase complex results in the accumulation of the initiating form of Pol II. (A) Western blot analysis of Pol II in lysates from 293T cells expressing the viral RNA polymerase trimer (3P) or the individual polymerase subunits PB1, PB2, or PA, or expressing no viral polymerase proteins (Control). Pol II was analyzed with the N-20 (Pol IIa and Pol IIo) or H14 (Pol II pS-5) antibodies. RanBP5 was detected as a loading control. The presence of the viral polymerase subunits was confirmed by Western blot analysis using antibodies specific for the individual polymerase subunits. The identity of bands is indicated on the right. Size markers in kDa are indicated on the left. (B) Quantitation of Western blots from panel A. Polymerase intensities were expressed as a percentage of intensities observed in the mock-transfected sample (Control) which was set to 100%. Two independent transfections were performed and each sample was analyzed twice by Western blot. The results shown, derived from four Western blot analyses, represent the average of two independent experiments with range indicated.

Next we asked the question whether the binding of the trimeric influenza virus RNA polymerase complex to the CTD of the large subunit of Pol II plays a role in triggering Pol II degradation in virus infected cells. We hypothesized that binding of the viral RNA polymerase complex to the CTD of the initiating form of Pol II could lead to Pol II pausing or arrest. This would trigger mechanisms analogous to those observed during DNA damage or in cells treated with α-amanitin when Pol II arrest is followed by the recruitment of the proteasome to Pol II leading to its ubiquitylation and degradation (Ratner et al., 1998; Somesh et al., 2005; Yang et al., 2003). We transfected 293T cells with plasmids expressing the PB1, PB2, and PA subunits of the viral RNA polymerase complex or performed transfections with a control plasmid (Fig. 4). When all three subunits were co-expressed, we observed a decrease in the non-phosphorylated unengaged (Pol IIa) and an increase in the phosphorylated transcriptionally engaged (Pol IIo) forms of Pol II. Western blot analysis of the serine-5 phosphorylated form of Pol II confirmed that at least some of the increased Pol IIo signal was due to the accumulation of the initiating form (Fig. 4). Interestingly, we also observed a decrease in the non-phosphorylated form in cells expressing the PA subunit alone, suggesting that the overexpression of PA is sufficient to induce the degradation of the non-phosphorylated form of Pol II. However, PA failed to induce an accumulation of the phosphorylated form, including the serine-5 phosphorylated form. Neither the expression of PB1 alone nor PB2 alone had a detectable effect on any of the Pol II forms (Fig. 4). Taken together, these results support the hypothesis that binding of the trimeric viral RNA polymerase to the CTD of Pol II induces Pol II arrest and its transient accumulation in a transcriptionally engaged form. This could result in the depletion of the transcriptionally unengaged form of Pol II as observed in cells expressing all three RNA polymerase subunits. However, the observation that the expression of the PA subunit alone can result in the decrease of the non-phosphorylated form of Pol II suggests that free PA, not associated with PB1 and PB2, could contribute to the depletion of the transcriptionally unengaged form of Pol II.

**Fig. 5:**
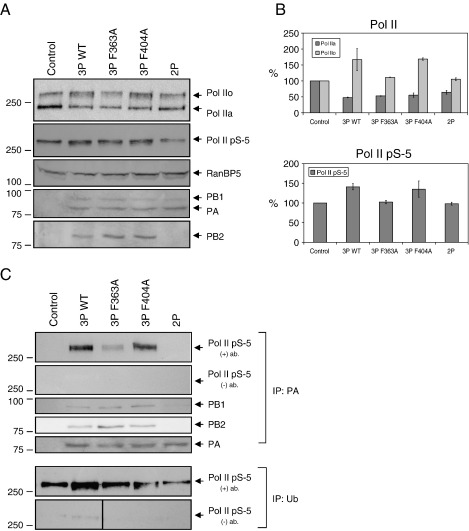
Binding of the trimeric viral RNA polymerase to Pol II is required for the induction of the accumulation of the initiating form of Pol II. (A) Western blot analysis of Pol II in lysates from transfected 293T cells expressing the viral RNA polymerase trimer [wild type (3P WT) or mutants with mutations in the PB2 subunit (3P F363A or 3P F404A)], a PB1-PA polymerase dimer (2P), or expressing no viral polymerase proteins (Control). Pol II was analyzed with the N-20 (Pol IIa and Pol IIo) or H14 (Pol II pS-5) antibodies. RanBP5 was detected as a loading control. The presence of the viral polymerase subunits was confirmed by Western blot analysis using antibodies specific for the individual polymerase subunits. (B) Quantitation of Western blots from panel A. Polymerase intensities were expressed as a percentage of intensities observed in the mock-transfected sample (Control) which was set to 100%. Two independent transfections were performed and each sample was analyzed twice by Western blot. The results shown, derived from four Western blot analyses, represent the average of two independent experiments with range indicated. (C) Immunoprecipitation of PA (upper panels) or ubiquitin (lower panels) from lysates of mock or virus infected cells. Immunoprecipitates with [(+) ab.] or without [(−) ab.] specific antibody were analyzed by Western blot using the H14 serine-5-specific Pol II antibody. Immunoprecipitates obtained with the PA antibody were also analyzed for the presence of the polymerase subunits PB1, PB2, and PA using specific polyclonal antibodies. Note the reduced levels of PB1 co-immunoprecipitating with PA in the absence of PB2 (2P).

In order to investigate whether the binding of the trimeric viral RNA polymerase to Pol II is a prerequisite for the accumulation of the transcriptionally engaged from, we took advantage of a PB2 mutant with reduced Pol II binding activity in the context of the trimeric viral RNA polymerase complex. This mutant, F363A, originally identified as a mutant deficient in cap-binding (Fechter et al., 2003) and subsequently found to have reduced Pol II-binding activity (Loucaides et al., 2009), showed a reduced induction of the accumulation of the transcriptionally engaged Pol IIo form (Figs. 5A and B). The serine-5 phosphorylated form of Pol II was not affected by the expression of this mutant RNA polymerase trimer either. In contrast, another PB2 mutant, F404A (Fechter et al., 2003), that retains wild-type levels of Pol II binding (Loucaides et al., 2009), induced a similar increase in Pol IIo, including the serine-5 phosphorylated form, as the wild-type trimeric RNA polymerase. Co-expression of PB1 and PA in the absence of PB2 (2P) resulted in no detectable effect on the Pol IIo and serine-5 phosphorylated forms of Pol II. In all cases when viral RNA polymerase subunits were expressed, a reduction in the Pol IIa form was observed, presumably due to the expression of the PA subunit. The Pol II-binding properties of the PB2 mutant polymerase complexes were confirmed by immunoprecipitation with a PA-specific antibody (Fig. 5C). Further immunoprecipitation experiments with an antibody specific for ubiquitin followed by Western blot analysis of the immunoprecipitates for Pol II showed that the accumulated serine-5 phosphorylated form of Pol II is ubiquitylated (Fig. 5C). Taken together, these results show that the accumulation of the ubiquitylated serine-5 phosphorylated form of Pol II is dependent on the binding of the viral RNA polymerase.

### Expression of the viral RNA polymerase results in the inhibition of Pol II transcription

**Fig. 6:**
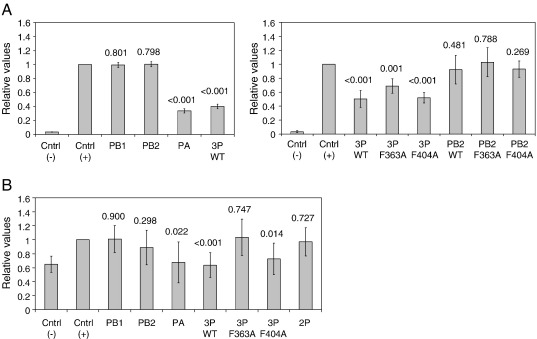
Expression of the RNA polymerase results in the inhibition of Pol II transcription. (A) Effect of viral RNA polymerase on IFN-inducible luciferase reporter gene expression. 293T cells were transfected with plasmids to express the indicated viral RNA polymerase subunits individually or in combination (see legend to Fig. 5 for details) and an IFN-inducible luciferase reporter plasmid. Luciferase expression was induced by IFN treatment and expression levels were determined by a luciferase assay. Luciferase levels in induced cells [Cntrl (+)] were set to 1. Cntrl (−), uninduced cells. Data presented are an average from 4 independent transfections, with standard deviations shown. (B) Effect of viral RNA polymerase on the IFN-inducible endogenous ISG15 gene. Transcription of the ISG15 gene was induced by IFN treatment and ISG15 mRNA levels quantitated by RT-PCR. mRNA levels in induced cells not expressing RNA polymerase subunits [Cntrl (+)] were set to 1. Cntrl (−), uninduced cells. Data presented are an average from 6 independent transfections with standard deviations shown. Two-tailed unpaired Student's *t* tests were performed to assess whether the values in the presence of RNA polymerase were significantly different from the values in their absence. The numbers shown above the bars represent the *p* values in comparison with Cntrl (+).

Our group previously demonstrated that Pol II elongation is inhibited in cells infected with influenza virus (Chan et al., 2006). The results described above suggest that this could be due to Pol II arrest induced by the viral trimeric RNA polymerase binding to the initiating Pol II and/or to PA targeting the non-engaged form of Pol II for degradation. To address whether the expression of the viral RNA polymerase complex inhibits Pol II transcription, we used an IFN-inducible luciferase reporter gene system (Didcock et al., 1999). We transfected 293T cells with a combination of plasmids to express the viral RNA polymerase subunits and a luciferase reporter and treated the cells with interferon. Cell lysates were analyzed for luciferase activity (Fig. 6A). IFN treatment resulted in a strong induction of luciferase activity [compare Control (+) to Control (−)]. The expression of individual PA or the trimeric polymerase complex resulted in a statistically significant (as assessed by a Student's *t* test) reduction in reporter gene activity, while the expression of individual PB1 or PB2 had no effect (Fig. 6A, left panel). In order to determine whether the binding of the trimeric RNA polymerase complex to the serine-5 phosphorylated form of Pol II contributes to the reduction, we used the PB2 F363A and F404A mutants described above (Fig. 6A, right panel). We observed a statistically significant reduction in reporter levels for both mutants although the reduction was less pronounced in the case of the F363A mutant suggesting that binding to Pol II might play a role. Neither of the individual PB2 mutants had a detectable effect on reporter levels.

We also analyzed the effect of the expression of the influenza virus RNA polymerase on an endogenous gene by taking advantage of the interferon-inducible ISG15 gene (Sadler and Williams, 2008). We transfected 293T cells with a combination of plasmids to express the viral RNA polymerase subunits as above and treated the cells with interferon to induce the ISG15 gene. We isolated total RNA and analyzed the levels of ISG15 mRNA by using quantitative RT-PCR (Fig. 6B). IFN treatment resulted in a relatively modest increase in the ISG15 mRNA. Neither the expression of PB1 alone nor PB2 alone affected the expression of the ISG15 mRNA. However, we found a statistically significant reduction in ISG15 mRNA expression when individual PA or all three RNA polymerase subunits (3P WT) were expressed. In contrast, we observed no statistically significant reduction in ISG15 mRNA levels when the wild-type PB2 was replaced with the F363A mutant that reduces the binding of the trimeric RNA polymerase to Pol II. The PB2 mutant with the F404A mutation that binds Pol II similar to the wild-type, inhibited ISG15 mRNA expression to the same level as the wild-type polymerase complex. The presence of PB2 was important for the inhibition of ISG15 mRNA expression as the PB1-PA dimer had no significant effect.

Taking the results from the two assays together, we conclude that the expression of individual PA or the trimeric viral RNA polymerase complex results in the inhibition of Pol II activity. The ability of the trimeric complex to inhibit Pol II appears to be affected by its Pol II-binding activity suggesting that the association between the viral and host transcriptional machineries is an important factor in the observed Pol II inhibition.

### Effect of Pol II inhibition and degradation on the regulation of viral RNA transcription and replication

Our results show that Pol II is inhibited and degraded in cells infected with influenza virus. However, influenza virus mRNA synthesis is absolutely dependent on Pol II activity as viral mRNAs are primed by capped RNA fragments derived from host mRNAs (Bouloy et al., 1978; Krug et al., 1979). The splicing of viral mRNAs and their nuclear export might also be dependent on active Pol II (Amorim and Digard, 2006; Amorim et al., 2007; Bouloy et al., 1978; Engelhardt and Fodor, 2006; Krug et al., 1979). How can one reconcile these apparently contradictory processes? How does the inhibition/degradation of Pol II affect the regulation of viral transcription and replication?

**Fig. 7:**
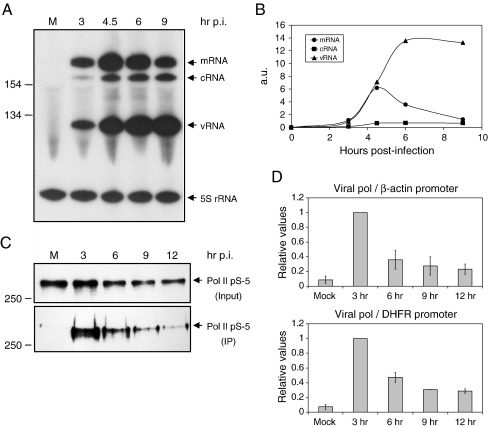
Shutdown of viral mRNA synthesis coincides with the degradation of Pol II. (A) Primer extension analysis of viral RNA levels in mock (M) and influenza A/WSN/33 virus infected 293T cells at the indicated time points post infection. The positions of viral mRNA, cRNA, and vRNA are indicated on the right. 5S rRNA was used as an internal control. Size markers in nucleotides are shown on the left. (B) Quantitation of RNA levels from panel A by phosphorimage analysis. (C) Immunoprecipitation of the viral RNA polymerase with a PA-specific antibody from lysates of mock (M) or virus infected cells. Input samples (upper panel, as shown in Fig. 1.) and immunoprecipitates (IP, lower panel) were analyzed with the H14 Pol II-specific antibody. (D) Analysis of the association of the viral RNA polymerase with Pol II promoter DNA during the viral life cycle by using ChIP. ChIP was performed using lysates from 293T cells either mock-infected or infected with influenza A/WSN/33 virus for the indicated periods of time and antibodies against the PA subunit of the RNA polymerase complex. Quantitation was performed by real-time PCR using primers specific for the promoter region of the β-actin or DHFR genes as described (Chan et al., 2006). Viral RNA polymerase densities were expressed relative to the sample isolated at 3 h post infection (maximal detected association) which was set to 1. An average of the data from two independent experiments is shown with range.

In order to address these questions, we investigated the accumulation of viral RNAs in infected cells at various times post infection. We infected 293T cells with influenza A/WSN/33 virus, isolated total RNA from infected cells at 3, 4.5, 6, and 9 h post infection, and analyzed viral RNA levels by a primer extension assay (Fig. 7A). Our results show that while maximal mRNA accumulation occurs relatively early during the replication cycle (between 3 and 4.5 h), vRNA synthesis continues late in infection (Fig. 7B). These results are in agreement with numerous previous studies reporting an early peak in mRNA synthesis, followed by exclusive vRNA synthesis late in infection (Hatada et al., 1989; Lee and Seong, 1998; Shapiro et al., 1987). Although several hypotheses have been put forward to explain this phenomenon (Perez and Donis, 1998; Watanabe et al., 1996; Ye et al., 1989; Zvonarjev and Ghendon, 1980), often referred to as a “switch” from transcription to replication, the precise molecular mechanisms involved remain unknown.

Our group proposed that viral mRNA synthesis requires the association observed between the viral RNA polymerase and Pol II as it might allow the viral transcriptional machinery to access the cap structure of nascent RNAs as well as RNA processing factors (Engelhardt et al., 2005). In order to investigate this association during the viral life cycle, we performed immunoprecipitations of the viral RNA polymerase using a PA-specific antibody and analyzed the immunoprecipitates for the presence of the initiating form of Pol II by using Western blotting (Fig. 7C). We observed that a maximal association between the viral RNA polymerase and Pol II occurs at 3 h post infection. At later time points, a much reduced association was observed. We also performed a ChIP assay of the association of the viral RNA polymerase with Pol II promoter DNA (Fig. 7D). We found maximal association with the β-actin promoter at 3 h post infection with dramatically reduced values at the later time points. Similar results were observed using the DHFR gene. The observation that the maximal association between the viral RNA polymerase and Pol II approximately coincides with the maximal viral mRNA accumulation suggests that the two processes might be linked providing support for the hypothesis that an association between the viral RNA polymerase and Pol II is required for viral transcription. These results also suggest that the dramatic downturn in mRNA synthesis that occurs at approximately 4.5 h post infection could be the result of the inhibition/degradation of Pol II induced by the accumulating levels of the viral RNA polymerase in infected cells.

## Discussion

In this paper, we show that the large subunit of Pol II is degraded in 293T cells infected with influenza A/WSN/33 virus. Degradation of Pol II was also observed in HeLa cells infected with A/WSN/33 (H1N1) (Chan and Fodor, unpublished), MDCK cells infected with SC35 or SC35M viruses (H7N7) (Gabriel and Fodor, unpublished), and 293T, COS-1, HeLa, and NLB2 cells infected with A/WSN/33 (H1N1) or A/Victoria/3/75 (H3N2) (Rodriguez et al., 2007), suggesting that Pol II degradation is a general phenomenon that can be induced by various subtypes of influenza virus. There is an increasing body of evidence that viruses have developed a multitude of strategies to inhibit host gene expression (Lyles, 2000). A general inhibition of host gene expression is likely to represent an efficient mechanism to suppress the activation of innate immune responses that are inevitably activated upon viral infection as a consequence of the expression of a variety of viral factors (e.g. 5′pppRNA, dsRNA) that are recognized by the host cell as foreign (Garcia-Sastre and Biron, 2006; Pichlmair and Reis e Sousa, 2007). Thus, influenza virus-induced Pol II degradation could represent a novel mechanism of inhibiting antiviral host responses.

How could influenza virus infection lead to the degradation of the large subunit of Pol II? We observed degradation of both the non-phosphorylated and phosphorylated forms of the large subunit of Pol II and there was a particularly severe decrease in the elongating (serine-2 phosphorylated) form from 6 h post infection. During viral infection, we detected the accumulation of Pol II forms recognized by an antibody specific for the serine-5 phosphorylated form that migrated at a higher than expected molecular weight. These results suggested that Pol II might be ubiquitylated and indeed, an immunoprecipitation using a ubiquitin-specific antibody, resulted in the detection of increased levels of ubiquitylated Pol II in infected cells. Ubiquitylation often targets proteins for degradation via the proteasome pathway (Weissman, 2001) suggesting that the proteasome might be involved in the degradation of Pol II. We attempted to use proteasome inhibitors (e.g. MG132, lactacystin) to investigate whether Pol II degradation could be prevented in influenza virus infected cells. Although we observed a delay in the degradation of Pol II, these results were inconclusive as the proteasome inhibitors also resulted in a delay in viral infection (Chan and Fodor, unpublished).

How is ubiquitylation of Pol II triggered in influenza virus infected cells? We speculate that binding of the viral RNA polymerase to the CTD of the initiating form of Pol II could trigger mechanisms that are known to be activated during DNA damage. DNA damage results in the arrest of Pol II at the DNA lesion often leading to the clearance of arrested Pol II by its degradation via ubiquitylation (Ratner et al., 1998; Somesh et al., 2005; Yang et al., 2003). Indeed, ChIP data show an increased association of Pol II with the DHFR promoter which is consistent with the idea of Pol II arrest. Upon the co-expression of the three subunits of the viral RNA polymerase complex, an accumulation of the serine-5 phosphorylated form of Pol II was also observed in the absence of viral infection. These results lend support to the hypothesis that transcriptional arrest triggered by the binding of the viral RNA polymerase to the initiating form of Pol II might be responsible for Pol II ubiquitylation.

Recently, Rodriguez et al. (2007) proposed that the proteolytic activity of the PA subunit of the viral RNA polymerase could be responsible for the degradation of Pol II in influenza virus infected cells. In particular, they showed that infection with a recombinant virus encoding a PA point mutant with reduced proteolytic activity resulted in a transient delay in the degradation of the hypophosphorylated from of Pol II. They also showed that the levels of hypophosphorylated Pol II were decreased in cells expressing the three subunits of the viral RNA polymerase, while expression of PA alone had no effect on Pol II levels. We confirmed that the levels of the non-phosphorylated form of Pol II were reduced upon the co-expression of the three viral RNA polymerase subunits, but surprisingly, overexpression of the single subunit PA resulted in a similar reduction of the non-phosphorylated form of Pol II. Thus, two mechanisms might operate during viral infection that could result in Pol II degradation: on one hand, individual PA, not complexed with PB1 and PB2, could induce the degradation of free unengaged form of Pol II; on the other hand, the trimeric polymerase complex by binding to the initiating form of Pol II induces its arrest and ubiquitylation that eventually lead to proteasome-mediated degradation. However, in virus infected cells, as opposed to transfected cells expressing PA alone, most of PA is likely to be present in complex with PB1 and PB2. Therefore, during viral infection, Pol II degradation induced by PA might not play a significant role and most Pol II degradation could be the consequence of Pol II inhibition induced by the binding of the trimeric viral RNA polymerase complex to the initiating form of Pol II.

We cannot, however, exclude the possibility that the proteolytic activity of PA also plays a role in inducing Pol II degradation in the context of the RNA polymerase trimer. It is possible that the association of PA with PB1 in the absence of wild-type PB2 (2P) or presence of a mutant PB2 (PB2 F363A) leads to a conformational change in PA that results in the inhibition of its proteolytic activity. Moreover, it should be noted that, in contrast to viral infections, no decrease in the transcriptionally engaged form of Pol II was detected in cells expressing the viral RNA polymerase subunits during the course of the experiment (up to 24 h post transfection) although the non-phosphorylated unengaged form was reduced. This suggests that for triggering degradation of the transcriptionally engaged form of Pol II, a viral factor not present in the transfected cells is required. We can only speculate that cleavage of the nascent host transcript by the viral RNA polymerase that is dependent on the presence of vRNA (Hagen et al., 1994) might be needed for the triggering of Pol II degradation. Removal of the 5′ cap structure of the nascent host transcript by the endonucleolytic activity of the viral RNA polymerase would lead to the exposure of the 5′ end of the transcript. Such transcript could be attacked by host nucleases, i.e. Xrn2, that play a role in the termination of Pol II after cleavage of the nascent transcript downstream of the poly(A) signal (West et al., 2004). We have attempted to address this question by reconstituting recombinant RNPs in 293T cells by transfecting plasmids to express the viral RNA polymerase subunits, NP, and a vRNA. However, we observed no reduction in the engaged form of Pol II possibly due to the low percentage of cells expressing functional RNPs (results not shown). Clearly, further studies are required to fully understand the molecular mechanisms of Pol II degradation in influenza virus infected cells.

Irrespective of the exact molecular mechanism, influenza virus-induced Pol II degradation inevitably affects the regulation of viral RNA synthesis. Previous studies suggested that influenza virus has developed a mechanism to regulate the synthesis of its own RNAs such that during the early stages viral mRNA is synthesized to allow viral protein production, while late in infection vRNA synthesis dominates to ensure sufficient levels of vRNA for the assembly of progeny virions (Shapiro et al., 1987). We confirmed previous results that viral mRNA synthesis peaks early during viral infection, while vRNA synthesis continues late in infection. However, our results suggest that the dramatic shut-off of viral mRNA synthesis late in infection might be the result of reduced interaction between the viral and host transcriptional machineries due to Pol II degradation. We found that maximal association between the viral RNA polymerase and Pol II occurred at about 3 h post infection, close to the time of maximal viral mRNA production. As infection proceeds, Pol II is degraded which results in the reduction of the association of the viral RNA polymerase with the Pol II transcriptional machinery as shown by co-immunoprecipitations and ChIP assay (see Fig. 7). In conclusion, we propose that the degradation of the large subunit of Pol II could be the cause for the reduction in viral mRNA synthesis late in infection. This contrasts the model by Rodriguez et al. (2007) who proposed that the degradation of the hypophosphorylated form of Pol II correlates with the onset of viral transcription.

We showed in this study that, in the presence of the PA subunit and the trimeric RNA polymerase complex that binds to Pol II, the expression of IFN-stimulated Pol II genes is inhibited, suggesting that Pol II inhibition induced by the viral RNA polymerase could play a role in the suppression of antiviral host responses. Another mechanism, proposed for inhibiting host gene expression and antiviral host responses involves the viral non-structural protein 1 (NS1) (Hale et al., 2008). NS1 was shown to interfere with host gene expression by inhibiting host mRNA polyadenylation via interactions with the 30-kDa subunit of the host cleavage and polyadenylation factor (CPSF30) and nuclear poly(A)-binding protein (PABII) (Chen et al., 1999; Nemeroff et al., 1998). More recently, the viral RNA polymerase has been proposed to form an integral component of the CPSF30-NS1 protein complex (Kuo and Krug, 2009). Thus, influenza virus might have developed a complex mechanism leading to host shut-off involving multiple viral components.

In summary, our findings suggest a novel mechanism for the inhibition of host gene expression in influenza virus infected cells. The viral RNA polymerase might play a crucial role in this mechanism by hijacking the host Pol II transcriptional machinery, eventually leading to the degradation of the large subunit of Pol II. Inhibition and degradation of Pol II will inevitably affect the expression of genes involved in antiviral host responses and therefore the pathogenicity of influenza viruses. While this study was under review, Rodriguez et al. (2009) provided experimental evidence that attenuated strains of influenza A viruses do not induce degradation of Pol II and proposed that the ability of influenza viruses to inhibit and degrade Pol II might contribute to their virulence. It remains to be determined to what extent RNA polymerases from various influenza virus strains, including highly pathogenic H5N1 viruses, differ in their ability to trigger Pol II inhibition and degradation.

## Materials and methods

### Cells and virus

Human embryonic kidney (293T) cells were obtained from the Cell Bank of the Sir William Dunn School of Pathology. Cells were cultured in minimum essential medium (MEM) supplemented with 10 % fetal calf serum (FCS) and 2 mM l-glutamine. Influenza A/WSN/33 virus was a gift from Dr. P. Palese (Mount Sinai School of Medicine, New York).

### Infections, transfections, and Western blot analyses

Infections were performed in 293T cells using influenza A/WSN/33 virus at an MOI of 4. Transfections were performed in 293T cells using Lipofectamine 2000 (Invitrogen) and plasmids encoding the RNA polymerase subunits of influenza A/WSN/33 virus (pCAGGS-PB1, pCAGGS-PB2, and pCAGGS-PA; gift of Dr. A. García-Sastre, Mount Sinai School of Medicine, New York). The F363A or F404A mutants of pCAGGS-PB2 were generated by site directed mutagenesis. Lysates from infected or transfected cells were analyzed by Western blotting using antibodies to detect the large subunit of Pol II (N-20, Santa Cruz), serine-5 phosphorylated CTD (H14, Covance), serine-2 phosphorylated CTD (H5, Covance), Pol III subunits RPC32 (clone C32-3) and RPC39 (clone C39-2) (Jones et al., 2000), karyopherin β3 (also known as RanBP5) (H-300, Santa Cruz), β-actin (Abcam), and the PB1, PB2, and PA viral RNA polymerase subunits (Carr et al., 2006; Deng et al., 2005). Signals were generated by using ECL reagent (Amersham or Millipore) and detected with a LAS4000 imager (Fuji) or by autoradiography. Images were quantitated using Aida and the results shown are an average of two independent experiments with range shown.

### Chromatin immunoprecipitation (ChIP)

ChIP was performed using a rabbit polyclonal PA antibody (gift of Dr. T. Toyoda, Shanghai Pasteur Health Research Foundation), a Pol II antibody (N-20, Santa Cruz), or a Pol III antibody (C32-3) (Jones et al., 2000) and lysates from 293T cells infected with influenza A/WSN/33 virus at an MOI of 4 as described (Chan et al., 2006). Mock-infected cell lysates and ChIP without a specific antibody served as negative controls. Quantitation was performed by real-time PCR using primers specific for the promoter region of the β-actin, DHFR, or 7SK RNA genes as described (Chan et al., 2006). Each experiment was performed twice and an average of the two experiments with range is shown.

### Immunoprecipitation assays

293T cells were infected with influenza A/WSN/33 virus at an MOI of 4, mock-infected, or transfected with the indicated combination of plasmids. Infected or transfected cells were harvested and resuspended in cell lysis buffer (50 mM Tris–HCl, pH 8.0, 200 mM NaCl, 1 mM MgCl_2_, 0.5% Igepal CA-630, 1 mM DTT, 25% glycerol, one Complete Mini EDTA-free protease inhibitor cocktail tablet (Roche)/10 ml) containing Benzonase nuclease (Novagen) (1 U/μl) and incubated for 1 h at 4 °C. Immunoprecipitations were performed using protein A-Sepharose CL-4B (Amersham) and antibodies against the PA subunit of the RNA polymerase (Dr T Toyoda) or ubiquitin (Abcam). Bound proteins were released by heating samples at 100 °C for 5 min in SDS-PAGE loading buffer.

### Pol II activity assay

In order to investigate Pol II activity in the presence of viral RNA polymerase, two assays (one employing a reporter gene and the other analyzing an endogenous gene) were used. In both assays, 293T cells were transfected with plasmids to express wild-type or mutant RNA polymerase subunits in different combinations, together with an interferon-responsive luciferase reporter plasmid [p(9-27)4tkΔ(-39)lucter kindly provided by Dr. R. Randall, University of St Andrews], as required. Twenty-four hours post-transfection, cells were treated with 1000 U/ml cell culture medium of Interferon-αA/D (Sigma) for 4 h. Luciferase expression was measured using a luciferase assay system (Promega) according to the manufacturer's instructions and a TriStar LB941 luminometer (Berthold Technologies). For the analysis of the endogenous gene, following interferon treatment total RNA was isolated using Trizol (Invitrogen) and mRNAs were reverse transcribed using a T_20_ primer and SuperScript II reverse transcriptase (Invitrogen). ISG15 cDNAs were quantitated by using real-time PCR and the following primers: 5′-GAAGGCGCAGATCACCCA-3′ and 5′-CTGCTGCGGCCCTTGTTA-3′. PCR was performed with the QuantiTect SYBR Green PCR Kit (Qiagen) and a Corbett Rotor-Gene RG-3000 cycler. Reactions were set up in triplicate, and data were analyzed by using the Comparative Analysis function of the Rotor-Gene 6 software.

### Analysis of viral RNAs by primer extension assay

293T cells were infected with influenza A/WSN/33 virus at an MOI of 4 or were mock-infected. Total RNA was isolated by using Trizol (Invitrogen) and primer extension analysis of the NA-specific viral RNAs was performed as described (Fodor et al., 2002; Vreede et al., 2004). Primer extension products were analyzed by polyacrylamide gel electrophoresis, detected by autoradiography, and quantitated by using a Fuji phosphorimager.
